# Relatives’ experiences of sharing a written life story about a close family member with dementia who has moved to residential care: An interview study

**DOI:** 10.1002/nop2.208

**Published:** 2018-10-04

**Authors:** Ewa Kazimiera Andersson, Helén Dellkvist, Ulrika Bernow Johansson, Lisa Skär

**Affiliations:** ^1^ Department of Health Blekinge Institute of Technology Karlskrona Sweden; ^2^ Karlskrona Municipality Karlskrona Sweden

**Keywords:** dementia, experiences, life story, person‐centred care, relative, residential care

## Abstract

**Aim:**

The aim of this study was to describe relatives’ experiences of sharing a written life story about a close family member with dementia who has moved to residential care.

**Design:**

An explorative descriptive qualitative design was used.

**Methods:**

The data were collected using semi‐structured interviews with a purposeful sample of eight relatives and analyzed using a qualitative content analysis.

**Results:**

Results show that creating and sharing the life story of a close family member could help relatives handle grief and stress. It was perceived as an important, yet difficult, task to ensure that the close family member got good quality care. The creation of a meaningful life story takes time and requires cooperation with family members and other significant people.

## 1 INTRODUCTION

Living with dementia means struggling to adjust to changes in life caused by the disease (Holmes, 2012). Age is the main risk factor for dementia, with prevalence increasing exponentially after the age of 60 and to approximately 20% at the age of 80. The number of incidents was estimated to be 35.6 million in 2010 and is expected to nearly double every 20 years, to 65.7 million in 2030 and 115.4 million in 2050 (Berr, Wancata, & Ritchie, 2005; Sosa‐Ortiz, Acosta‐Castillo, & Prince, 2012). The growing group of people posing thereby a great challenge for the future of healthcare (United Nations, 2015). Dementia is a general term for a syndrome including a range of chronic or progressive organic brain diseases that are characterized by difficulties of short‐term memory often followed by non‐cognitive symptoms, referred to as behavioural and psychological symptoms of dementia (Song & Oh, 2015; World Health Organization [WHO], 2016). It is known that the use of life story (LS) can enhance person‐centred care for people with dementia by enabling the healthcare professionals to know each person's needs, preferences and to see the person behind the illness (McKeown, Clarke, Ingleton, Ryan, & Repper, 2010). Relatives are those significant persons who can tell and create the LS about a close family member during admission to residential care (Hennings, Froggatt, & Payne, 2013). Knowledge of this nature is needed to understand what should be prioritized in the development of dignified and respectful care for people with dementia.

## 2 BACKGROUND

People living with dementia have increased vulnerability, which can influence their daily life in a negative way as they may lack understanding from people around them. Living with dementia also makes it difficult for the older person to express and communicate their needs (Miyamoto, Tachimori, & Ito, 2010). Relatives might therefore be of importance telling their LS about a close family member for healthcare professionals during admission to residential care (Karlsson, Sävenstedt, Axelsson, & Zingmark, 2014). A LS can be expressed as a written text with information about the person, photographs and/or personal belongings significant for the person (Eley & Kaiser, 2017). It should include some evaluative points, which communicate moral values of the person and events that have had a special meaning that can be told and retold throughout life (Linde, 1993). The LS may contain events of significant memories from the person's life (McKeown et al., 2010). A LS mirrors in that way identities of people's lives by putting together things, past actions and events, into a meaningful LS and the person's dignity can thereby be preserved (Polkinghorne, 1996). When people are in the midst of their LS, which is true for people living with dementia, both the past and the future become important. What has happened to them in the past cannot be changed, although future events may change the importance of certain events from the past and the thoughts of how the rest of their life will be.

A LS, furthermore, supports the healthcare professionals in communicating with the person living with dementia and makes it possible to get to know the person and interpret and understand the person's needs and desires (Edvardsson, 2010; McKeown et al., 2010). Research (Heggestad & Slettebø, 2015; McKeown et al., 2010) shows that active use of a LS approach is a mechanism to improve person‐centred care in residential care. A LS makes it also possible for healthcare professionals to see the person behind the disease, support the person in being seen and heard and help the person feel safe. However, as a relative to write down and share a LS about a close family member can be difficult, as the relative has to decide what to include and exclude, as some things may be too sensitive or hurtful to include. A trustful relationship must therefore be established with the healthcare professionals so they can support the relatives in this process (Ericson, Hellström, Lundh, & Nolan, 2001). Healthcare professionals may also help relatives handle complex emotions that come up when writing the LS (Kellett, Moyle, McAllister, King, & Gallagher, 2010). According to the literature review, it becomes obvious that relatives are an important source of knowledge when a LS is being created during admission to residential care. Focusing relatives’ experiences about sharing a written LS about a close family member during admission to residential care may be used as knowledge for healthcare professionals to improve person‐centred care. The aim of this study was to describe relatives’ experiences of sharing a written LS about a close family member with dementia who has moved to a residential care.

## 3 METHODS

### 3.1 Design

An explorative qualitative design was chosen. Data were collected with semi‐structured interviews (Polit & Beck, 2012) and analysed using content analysis (Graneheim & Lundman, 2004).

### 3.2 Participants and procedure

A purposeful sample of eight relatives participated in the study. The inclusion criteria were as follows: (a) being a relative to a close family member with dementia in a residential care, (b) having written a LS about the person and shared it with the healthcare professionals in the residential care in the last 6 months and (c) being informed by a registered nurse specialized in dementia (RND) about how to write a LS by using a template. There were five daughters, one son, one husband and one wife. The participants were between the ages of 52‐72 years (mean = 61). Four participants had a university education, four a high school education. Three of the participants were retired, and the others were working.

A contact person at a Research Centre forwarded an information letter to RND working in five municipalities in a rural area of southern Sweden, to support the recruitment process. The RND gave eligible relatives an information letter with a consent form. Nine accepted the invitation, but before the interview took place, one of them withdrew.

### 3.3 Data collection

Between March and April 2016, individual semi‐structured interviews were conducted using an interview guide. This design was chosen to obtain data as detailed and complete as possible and to give the participants the opportunity to speak specifically about their experiences (Polit & Beck, 2012). The interviews were based on the following four open‐ended questions: (a) How would you describe your experiences of sharing your close family member's LS? (b) Did you experience any difficulties when you shared your close family member's LS? (c) Did you experience any opportunities when you shared your close family member's LS? (d) What were your thoughts and feelings when you shared the LS of your close family member? The questions were followed by probing such as: Could you explain further?, What do you mean?, Please tell me more. A pilot interview was conducted to test the suitability of the questions and get experiences in interviewing. The interviews lasted for about 40 min and were digitally recorded and transcribed verbatim. The participants were interviewed in different places according to their preference.

### 3.4 Data analysis

To analyze the interviews the researcher used a content analysis (Graneheim & Lundman, 2004). The transcripts of the interviews were read and re‐read several times to get a sense of the whole text. The text was then divided into meaning units. In content analysis, an important decision is the selection of meaning units to be analyzed—that is, sentences or phrases that are related to the aim. Each meaning unit was then condensed and coded—that is, the text was shortened and labelled with a code that described the content. The codes were then compared and sorted into subcategories based on differences and similarities. The analytical process was characterized by moving back and forward between the whole text and the parts of the texts. All authors discussed the meaning units, the condensation, the codes and finally validated the categories in the original text. According to Graneheim and Lundman (2004), creating categories is the core feature of qualitative content analysis.

### 3.5 Ethical consideration

All the participants were given verbal and written information about the study and were told that participation was voluntary. They were assured of confidentiality and the right to withdraw from the study without any explanation. The study was conducted in compliance with the established ethical guidelines of the Declaration of Helsinki and received ethical guidance from the Ethical Advisory Board in South‐East Sweden (No. 330‐2016).

## 4 RESULTS

The analysis resulted in four categories: The establishment of a LS is a way to process grief and stress; Be the voice of the close family member; Need to understand the meaning of a LS; and Lacks a creative design for the LS. The categories are presented in the text below and illustrated with quotations from the interview texts to verify the categories.

### 4.1 The establishment of a LS is a way to process grief and stress

The relatives described emotions of grief and stress associated with the progression of the dementia, which made it difficult to write and share the LS of their close family member with the healthcare professionals. They described their own life situation as intolerable since they had been forced to take full responsibility for the close family member's everyday life before the move to the residential care. The relatives experienced stress, fatigue and exhaustion due to the psychological strain that they had lived with for a long time. They also felt emotionally influenced because of the new and dazing situations they had to handle during their close family member's transition to residential care. To write a LS in this situation was experienced as too much to deal with. The relatives experienced that they first needed to adjust to the new life situation and handle their stress and grief before they could manage to write and share a LS of their close family member. One participant stated:

… it was so much with kind of everything then, then when she came there and should move to an accommodation and it was so turbulent before and everything, so you were pretty exhausted as a relative ….

Sharing a written LS was also experienced as a possibility to deal with the grief that came out of the separation caused by the dementia and the close family member's transition to the residential care. The relatives experienced that the writing of a LS was a way to express their own feelings, to look back on and remember their previous life together with the close family member and reach conclusions. To write and share the LS was therefore a way to accept the situation as well as to get an integrated picture of their past life together. One participant stated:

maybe this has been some kind of therapy for me, to describe what our life has been like, I have many times felt that, yes, the possibilities for me to go on living in a way has been to think about our earlier life together.

### 4.2 Be the voice of the close family member

The relatives described that it was important to be the voice of the close family member but that they needed time for the task. It took a lot of time to write down the LS and at the moment they lacked time due to the care duties of the close family member's dementia progression, which sometimes was impossible to manage. A lot of things had to be taken care of in addition to the personal care of the close family member, such as documents, bills and several contacts with different authorities. The lack of time also limited their possibilities to investigate their close family member's previous life and write down the LS. They experienced that they had to be committed to the task and could not write down the LS in haste. The relatives explained that they needed more time to investigate details about the close family member's life and carefully choose relevant information. One participant stated:

…then I realized that it wasn´t just to write it down…I really wanted to do it and then I felt that I hadn´t got the time…that if I should do a story it required some research and a commitment that I didn´t feel like I had right then….

It was important to write a suitable LS—one that the close family member could feel proud of. The relatives experienced that writing the LS was difficult because some parts of the close family member's life were unknown to them, but they thought that some of these parts could be of relevance for the healthcare professionals. Therefore, they had to create the LS by putting pieces together in the form of text, photos and letters. In this process, the relatives experienced a need to consult other family members and significant people to get a complete picture of the close family member. The relatives experienced that it was positive to sit down with siblings or parents to remember and share memories from the past and together create the LS. Some relatives said that they included conversations with other family members who told stories about the person's life history, thereby making the LS richer and mirroring significant people's voices:

…a resumé of thoughts, different experiences among the siblings…yes we shared, stories and situations…

The relatives felt that if they were given the possibility and had time to investigate their close family member's life history, the LS could be more detailed and informative and thereby be more useful in daily life and contribute more to better quality of care. The relatives wished that the LS would have been written in an earlier stage of the dementia, as they thought it would be more beneficial in the daily care of their close family member. They described also the importance that the close family member then would be able to take part in creating the LS's content which could help them to recognize themselves.

### 4.3 Need to understand the meaning of a LS

The relatives described that it was important to understand the meaning of a LS but felt that the written information they received from the municipal officials about the importance was insufficient. Some of them expressed that when information about the LS was submitted together with other information and documents from the municipal office, there was a risk that information was lost in the wealth of information provided. Also, the time at which the information was given was of importance for the possibility of writing a good LS. The relatives experienced that it was difficult to take in so much information in this new and bewildering life situation. They experienced a constant uncertainty about the information they had been given about how to write a LS. They felt that even though the written information had been provided, they did not completely understand it. The relatives described that it would be of importance that the professionals who provided the information ensure that the message had been understood and they requested more opportunities to access information.

Receiving verbal information from the healthcare professionals, especially about the importance of writing a LS and filling in the template at the same time, was positively experienced. It was important to have the possibility to ask questions. One relative described how a registered nurse both gave information and invited the relative to a lecture about the importance of writing a LS. The information and the lecture were perceived to be both clear and inspiring, which motivated the relative to think outside the box and create a LS without filling in the existing template:

…she made me go on an information about LS … she described why LS was important and how it was a way to take care of the people in residential care and I probably thought it was good that I, I did it before I received any request from the municipality…then I have done it in my way…I wrote my own without following any template….

The relatives understood that the LS was a working tool for healthcare professionals and that it would help to provide security and good care for their close family member. They expressed a strong desire that healthcare professionals, through the LS, would know the close family member better and understand who the person was before the illness. It was considered important that healthcare professionals knew what the close family member had liked and not liked earlier in life to be able to deliver good care. The LS was helpful to facilitate communication between them as relatives and healthcare professionals since the close family members could not always understand what was being said or express themselves in words. However, the relatives were unsure whether the healthcare professionals using the LS after it had been written and submitted to them.

### 4.4 Lacks a creative design for the LS

The relatives expressed a wish that the LS template that they received were designed differently. They felt that the existing template was too long, too stilted, too complicated or that there were too few lines to write on. They experienced a need to simplify and adapt the existing template to each person's needs and preferences. They suggested that the LS should include images, written stories, songs or other material that showed what the close family member enjoys and finds pleasure in. The relatives expressed that the only limitation in the design of a LS is your own imagination. They described that designing the LS based on their close family member's needs would make it easier for healthcare professionals to know and understand who the person once had been and, thereby, support the daily care of their close family member:

…sometimes it was difficult to answer some questions because how detailed should you be… I think you have, it may not be too long…Maybe it was a bit many pages … think you should keep it simple…

The relatives experienced further difficulties in answering questions in the existing template. It was sometimes difficult to understand the issues and to select what the LS should include, and it was difficult to know how much text should be written under the headings. The relatives were not sure if the healthcare professionals would have the time and dedication to take part and learn from the LS. As an opportunity for development of the template, the relatives proposed that the close family member's contact person in the residential care could interview them and help write the LS. For the healthcare professionals, this would mean that they got the information they needed for daily care and for the relatives it would mean a relief to not have to think about the issues that they felt difficult to answer.

The relatives experienced that the existing template, despite its limitations, still gave the opportunity to take up what felt most important. Several relatives wrote about major life events, such as the close family member's childhood, family, professional life, interests and ageing. Among the most important things for the relatives to not write down was anything that would hurt, be negative or harm the close family member. It felt important not to leave out the close family member in the writing of the LS:

…you would never write about the bad things, you don't, you only write about the good things and what's good for the staff to know… you don't want to leave them (close family member) out….

## 5 DISCUSSION

The results from our study show that writing a LS describing a close family member with dementia moving from the home to a residential care was a difficult process filled with stress and grief. The relatives described that it was important to be the voice of the close family member but felt that they need sufficient time for the task as well as more detailed information about how to write and share a LS by answering questions in the existing template. According to Helgesen, Athlin, and Larsson (2015) relatives of people living with dementia are often stressed, as they are responsible for the person's care, which may be experienced as a burden. Being responsible is a physical and psychological stress that causes exhaustion (Graneheim, Johansson, & Lindgren, 2014). Progression of the dementia causes impairments and that leads to the close family members not being able to take care of themselves, their actions or express their will. Relatives seeing the person being changed may feel grief and become psychologically ill (Helgesen et al., 2015).

Sharing the LS can be a way to process the grief over the changed life situation and accept the separation from the close family member. Research (Kellett et al., 2010) describes relatives’ experiences of remembering essential events and moments in terms of a renewed strength and of seeing the earlier life as rich and meaningful. The renewed strength made it easier for the relatives to accept the changed situation, which may also have reduced the stress and grief. For a moment, it was possible to forget about the dementia, stand by the side and remember the close family member as the person he/she once was. Relatives feel increased satisfaction by sharing the LS and they feel confirmed and sense that the healthcare professionals value them (Karlsson et al., 2014).

Relatives in our study reported that it was important to be the voice of the close family member. However, the personal commitment required to write down the LS was felt to be too much. The relatives were responsible for practical matters, such as paperwork and official contacts due to the transition to the residential care, which limited their time and energy for LS‐writing. Research (Egan et al., 2007) shows that it is difficult for relatives to share a LS about someone else if there is lack of time. Creating a LS means to investigate someone else's life with personal details sometimes unknown for the relative. Therefore, it is an advantage to start writing down a LS early in the stage of dementia, when the close family member can participate (Karlsson et al., 2014). This increases the chance that it is the close family member living with dementia's LS and not an incomplete interpretation made by a relative. In the absence of knowledge about the close family member's life, relatives tend to make assumptions based on the insufficient knowledge they have (Egan et al., 2007). Unfortunately, this often results in a blank and predictable story, both impersonal and inaccurate. A well‐written LS on the other hand is one of the most important tools to share and represent the close family member's history and identity.

The result shows further, the importance that the LS describes the family member's personality and earlier life in a proper way, so he/she could receive good care. Person‐centred care affirms the identity of the person despite the impairments due to the dementia. The person living with dementia can therefore keep a feeling of identity and the ability to communicate needs and wishes would be maintained (Stein‐Parbury et al., 2012). Kellett et al. (2010) stated that the written LS is an aid for professionals in personalizing the nursing care, which can lead to an understanding of the person through the LS and helps maintain their identity. A LS may also support healthcare professionals to understand what brings meaning to the life of the person living with dementia, which also the relatives in this study pointed out. Furthermore, a LS helps healthcare professionals stay focused on the person living with dementia, learn from their life and listen, respond and deal with the symptoms the person expresses (McKeown et al., 2010; Resnick, 2017).

The relatives in our study experienced a need to consult other family members to fill knowledge gaps about the close family member's earlier life. They had positive experiences of sharing memories with the close family member but needed support from other family members to be able to write an extensive LS. Thompson (2011) believes that a person owns his own life history and the written LS should, as far as possible, be designed as the person living with dementia wants and expresses. But severe cognitive impairment can make it necessary for a relative to give a helping hand with the LS. After that, the LS can develop and follow the person when moving into a residential care. It is, therefore, according to Thompson (2011), important that a LS should be written in an early stage of the dementia, so it can be of use and help for the person living with dementia, the relatives and healthcare professionals. The results in our study show that the LS were asked for too late, when the person living with dementia had already moved into the residential care. However, when relatives write the LS together with the person living with dementia it may also lead to an improved relationship and reduced negative feelings (Subramaniam, Woods, & Whitaker, 2014). By creating the LS together, the relatives may bond with the close family member and re‐establish contact. It may also contribute to a meaningful communication and quality time between them (Russell & Timmons, 2009; Subramaniam et al., 2014). A LS may be a joy to descendant relatives through positive experiences in making copies of the LS, through giving copies to other family members for remembrance and through reading the LS about the close family member (Subramaniam et al., 2014). Thompson (2011) implies that a written LS leads to an increased understanding, a deeper sense of belonging. This gives feelings of safety, reduces anxiety and improves the relationship between the relative, the close family member living with dementia and the healthcare professionals.

Our results show that relatives needed to understand the meaning of a LS and what facts they should have included. However, some of them lack information about when to create a LS and experienced that much information was handed over at the same time, which made it difficult to deal with. The relatives felt unsure and stressed if they received information they did not understand. Kellett et al. (2010) describe how relatives experience confusion, aimlessness and uncertainty when it comes to writing a LS. They are not sure what they are supposed to write, and they do not know which information is important or relevant. Relatives need, therefore, to communicate the LS during the creative process with both the person living with dementia, other family members and healthcare professionals. A well‐written LS enables the healthcare professionals to see the person behind the disease, which makes the nursing care more person‐centred and this leads to improved relationships (Russell & Timmons, 2009).

Relatives in our study described that the template they received was too long, stilted and difficult to fill in and therefore requested a different design of the LS template and wanted it to be written based on the needs of their close family member. Research (Thompson, 2011) shows that a LS can be designed in many ways—for example, as a book, leaflet, collage, memory box or electronically. However, it is important to choose a format that is suitable for all involved. The written LS is often more detailed when described and presented as a book, while a collage is more like a creative project about the person living with dementia and their relatives. Furthermore, memory boxes contain subjects that are of value to the person living with dementia. Clarke, Hanson, and Ross (2003) stated that a combination of written text, personal stories and pictures, often of significant people or events, make the LS alive and can elicit memories. Photos can also facilitate communication and create a stronger bond between the person with dementia and healthcare professionals.

### 5.1 Strengths and limitations

This study has limitations that need to be discussed. First, the sample size in this study was quite small but according to Sandelowski (1995), the sample size in qualitative research should be large enough to achieve variations in experiences and small enough to permit a deep analysis of the data. The present sample with eight relatives was considered appropriate to maintain depth in the analysis. The participants were also recruited by means of a purposeful sampling, which means that they were selected based on their special knowledge about the studied phenomenon. The included participants had a high educational qualification and that fact may be regarded as a possible selection bias (c.f. Polit & Beck, 2012). However, the level of education was not an inclusion criterion. The participants gave rich descriptions of their experiences and as a result, their stories contained variations of the phenomenon under study. Dependability was ensured as an interview guide was used, and follow‐up questions were integrated, which allowed the interview to be more reflective. This data collection method decreased the risk of inconsistency (Polit & Beck, 2012). To achieve trustworthiness of the analysis, the authors discussed the findings until consensus was reached, which strengthens the credibility of the study. The results cannot be generalized as they are limited to the small sample size but can be transferred to similar situations if the results are re‐contextualized (Polit & Beck, 2012).

## 6 CONCLUSION

This study indicates that it is important for relatives to share a close family member's LS with healthcare professionals at the residential care, but at the same time, it is a stressful process that should start as early as possible when a person got a dementia diagnosis. To write and share the LS was experienced as being important in ensuring that the close family member got good quality care and was also a way to handle their own grief and stress over the changed life situation when the close family member moved to the residential care. It was difficult for relatives to share a LS about the close family member, as creating a LS means investigating someone else's life with personal details sometimes unknown to them. However, to create a useful LS means to have enough time and possibilities to include significant people, places, personal things and memories in a creative design to maintain a positive view of the close family member's life. Registered nurses need to involve close relatives to contribute with important information to the LS and thereby preserve personhood and enhance the dignity when a person with dementia is admitted to residential care. A LS is useful and important for the nursing staff to provide person‐centred care based on the individual's needs and resources.

## CONFLICT OF INTERESTS

No conflict of interests have been declared by the authors.

## AUTHOR CONTRIBUTIONS

All authors contributed to design the study, HD and UBJ collected the data. EKA, HD and UBJ analyzed the data. All authors together drafted the manuscript and contributed to editing the final manuscript, revised it critically for scientific content and approved the final version.
